# Randomized Clinical Trials of Gene Transfer for Heart Failure with Reduced Ejection Fraction

**DOI:** 10.1089/hum.2016.166

**Published:** 2017-05-01

**Authors:** William F. Penny, H. Kirk Hammond

**Affiliations:** ^1^ VA San Diego Healthcare System, San Diego, California.; ^2^ Department of Medicine, University of California, San Diego, San Diego, California.

**Keywords:** gene therapy, SERCA2a, stromal cell-derived factor-1, adenylyl cyclase type 6

## Abstract

Despite improvements in drug and device therapy for heart failure, hospitalization rates and mortality have changed little in the past decade. Randomized clinical trials using gene transfer to improve function of the failing heart are the focus of this review. Four randomized clinical trials of gene transfer in heart failure with reduced ejection fraction (HFrEF) have been published. Each enrolled patients with stable symptomatic HFrEF and used either intracoronary delivery of a virus vector or endocardial injection of a plasmid. The initial CUPID trial randomized 14 subjects to placebo and 25 subjects to escalating doses of adeno-associated virus type 1 encoding *sarcoplasmic reticulum calcium ATPase* (AAV1.*SERCA2a*). AAV1.SERCA2a was well tolerated, and the high-dose group met a 6 month composite endpoint. In the subsequent CUPID-2 study, 243 subjects received either placebo or the high dose of AAV1.*SERCA2a*. AAV1.*SERCA2a* administration, while safe, failed to meet the primary or any secondary endpoints. STOP-HF used plasmid endocardial injection of *stromal cell-derived factor-1* to promote stem-cell recruitment. In a 93-subject trial of patients with ischemic etiology heart failure, the primary endpoint (symptoms and 6 min walk distance) failed, but subgroup analyses showed improvements in subjects with the lowest ejection fractions. A fourth trial randomized 14 subjects to placebo and 42 subjects to escalating doses of adenovirus-5 encoding *adenylyl cyclase 6* (Ad5.*hAC6*). There were no safety concerns, and patients in the two highest dose groups (combined) showed improvements in left ventricular function (left ventricular ejection fraction and –dP/dt). The safety data from four randomized clinical trials of gene transfer in patients with symptomatic HFrEF suggest that this approach can be conducted with acceptable risk, despite invasive delivery techniques in a high-risk population. Additional trials are necessary before the approach can be endorsed for clinical practice.

## Introduction

Heart failure (HF) affects six million patients in the United States.^1^ Although there has been some reduction in the HF admission rate and mortality in the last 10 years, the prognosis and burden of HF remains unacceptably high. New therapies are needed, which is the rationale for considering new approaches such as gene transfer.

Although many preclinical studies have shown benefits of gene transfer in animal models of HF, few have been translated to randomized clinical trials. The attraction of gene transfer is its simplicity—a one-time administration offers the possibility of safely providing benefits beyond standard pharmacological and device therapy in this devastating disease with high morbidity and mortality.

Gene transfer to improve the function of the failing heart in patients with HF with reduced ejection fraction (HFrEF) has now been performed in four randomized, placebo-controlled clinical trials. Three strategies have been employed: (1) intracoronary (IC) delivery of adeno-associated virus type 1 encoding *sarcoplasmic reticulum Ca2+-ATPase* (AAV1.*SERCA2a*); (2) endocardial injection of a plasmid encoding *stromal cell-derived factor-1* (*pSDF-1*); and (3) IC delivery of adenovirus-5 encoding *adenylyl cyclase type 6* (Ad5.*hAC6*). These approaches, used in four randomized clinical trials, are the focus of this review.

## Methods

PubMed and ClinicalTrials.gov (without language restrictions) were searched for clinical trials published between January 1990 and October 2016, using the search terms “gene transfer” (and “gene therapy”) and “heart failure” and related terms. Only four randomized clinical trials testing gene transfer in patients with HF were found, confirming that gene transfer for clinical HF is in its infancy. These four trials are summarized by providing the rationale for transgene selection, important preclinical or nonrandomized clinical trials, safety, and results. Finally, the results of the studies are summarized and perspectives are provided for future work in this area.

## Results and Discussion

The CUPID^2^ and CUPID-2^3^ trials used IC delivery of AAV1.*SERCA2a*. SERCA2a is a cardiac Ca2+ regulating protein. CUPID-2 used simultaneous intravenous infusion of nitroglycerine during IC vector delivery. The STOP-HF^4^ clinical trial used endocardial injection of pSDF-1 to promote stem-cell recruitment and tissue repair, including angiogenesis in patients with HFrEF of ischemic etiology. Finally, a fourth randomized clinical trial used IC delivery of an adenovirus-5 vector encoding adenylyl cyclase 6 (Ad5.hAC6).^5^ The AC6 gene transfer trial used simultaneous IC infusion of sodium nitroprusside during IC vector delivery (Table 1).

**Table 1.** Features of randomized clinical trials of gene transfer for heart failure

	*Vector*				*Population*					*Protocol*		
*Trial (Phase)*	*Gene*	*Vector (promoter)*	*Delivery*	*Dose (*n*)*	*Group size*	*Ischemic (%)*	*EF (%)*^a^	*NY class% 2–3–4*	*% Screen failure*	*Primary endpoint*	*Secondary endpoint*	*Duration (months)*
CUPID^2^ (P2)	*SERCA2a*	AAV1 (CMV)	IC + IC NG bolus or IV NG infus	6 × 10^11^ drp (8) 3 × 10^12^ drp (8) 10^13^ drp (9)	Rx: 25 Plac: 15	49	25 ± 2	0–100–0	92	Composite: symptoms, exercise, BNP, echo, clinical outcomes	Days after transplant,^b^ VAD, HF hospitalization, death; EDV	12
CUPID-2^3^ (P2b)	*SERCA2a*	AAV1 (CMV)	IC + IV NG infus	10^13^ drp	Rx: 121 Plac: 122	63	24 ± 1	18–81–1	84	Time to worsening HF (hosp or outpatient)	Time to first event: death, transplant, VAD	12
STOP-HF^4^ (P2)	*SDF-1*	Plasmid (CMV)	Endocardial injection	15 mg (32) 30 mg (20)	Rx: 62 Plac: 31	100	28 ± 1	32–67–1	53	6 min distance + symptoms (composite)	Echo (EF, volume)	12
AC6^5^ (P2)	*AC6*	Ad5–E1/E3 (CMV)	IC + IC NP infus	3.2 × 10^9^ vp (6) 3.2 × 10^10^ vp (6) 10^11^ vp (6) 3.2 × 10^11^ vp (12) 10^12^ vp (12)	Rx: 42 Plac: 14	48	31 ± 1	45–51–4	35	EF ETT time LV dP/dt	Hemodynamics Symptoms ICD events	12

^a^ Mean baseline EF (±*SE*) of randomized subjects.

^b^ Days after transplantation or ventricular assist device (VAD), hospitalization, or not alive.

P, phase; CMV, cytomegalovirus promoter-enhancer; *SERCA2a*, sarcoplasmic reticulum calcium ATPase; *SDF-1, stromal cell-derived factor-1*; *AC6, adenylyl cyclase type 6*; AAV1, adeno-associated virus type 1; Ad5–E1/E3, human E1/E3-deleted adenovirus-5; IC, intracoronary; IV, intravenous; infus, infusion; NG, nitroglycerine; NP, sodium nitroprusside; drp, DNAse resistant particles; vp, virus particles; Rx, received vector; Plac, received placebo; NY Class, NYHA symptom classification; ETT, exercise treadmill test; Echo, echocardiography; EF, ejection fraction; LV, left ventricular; EDV, end-diastolic volume; HF, heart failure; dP/dt the rate of LV pressure change; ICD, implantable cardiac defibrillator.

### CUPID and CUPID-2 (AAV1.*SERCA2a*)

#### Preliminary preclinical and non-randomized clinical studies

SERCA2a is a Ca2+ regulating protein on cardiac myocytes. Isolated cardiac myocytes from patients with HF have reduced expression and activity of SERCA2a and reduced shortening, abnormalities that are rectified by *SERCA2a* gene transfer.^6^ Intracoronary delivery of AAV1.*SERCA2a* (10^12^ DNAase resistant particles, drp) in pigs with volume overload was beneficial and was associated with increased cardiac SERCA2a expression.^7^ In an initial open-label clinical trial (not randomized), nine subjects with stable symptomatic HFrEF (EF ≤30%) received IC AAV1.*SERCA2a* (1.4 × 10^11^, 6 × 10^11^, or × 10^12^ drp). Follow-up for 12 months after infusion showed no serious adverse events and evidence of improvement in symptoms, exercise, left ventricular (LV) function, and biomarkers.^8^ These data were the basis for two subsequent randomized clinical trials.

### CUPID

The CUPID trial^2^ was a Phase 2 blinded, randomized, placebo-controlled, multicenter study examining the effects of AAV1.*SERCA2a* in 39 subjects with stable symptomatic HFrEF (EF ≤35%), maximal oxygen consumption ≤20 mL/kg/min, and an implantable cardioverter defibrillator (ICD). Fourteen participants received IC placebo, and 25 received IC AAV1.*SERCA2a*, at doses of 6 × 10^11^ (*n* = 8), 3 × 10^12^ (*n* = 8), or 3 × 10^13^ (*n* = 9) drp. Subjects were screened to exclude those with detectable neutralizing antibodies against AAV1. The trial used five efficacy domains: symptoms, functional (6 min walk distance and peak oxygen consumption), biomarker, LV volume and ejection fraction, and clinical outcomes, which included seven different endpoints.

#### Safety in CUPID

IC AAV1.*SERCA2a* was well tolerated, with no untoward effects attributed to the vector or transgene 1 year later. The incidence of serious and non-serious adverse events was highest in the subjects who received placebo and lowest in those who received the highest dose of AAV1.*SERCA2a*. There was no evidence of cellular immune response to the vector. There were eight deaths: four in the placebo group, three in the low-dose, one in the mid-dose, and none in the high-dose groups. The differences in mortality were not significant (Table 2).

**Table 2.** Results of randomized clinical trials of gene transfer for heart failure

		*Efficacy*			
*Trial*	*Safety (SAE rate vs placebo)*	*Primary endpoint*	*Secondary endpoint*	*Other*	*Status*
CUPID^2^	No difference vs. placebo	Composite endpoint met, *p* < 0.2, which was pre-specified as “significant”^b^	Decreased LVEDV (*p* = 0.01) Reduced CV event risk (*p* = 0.003)	Not applicable	Precursor to CUPID-2
CUPID-2^3^	No difference vs placebo^a^	Primary endpoint not met	Secondary endpoint not met	Not applicable	No further HF trials planned
STOP-HF^4^	No difference vs placebo	Primary endpoint not met	Patients with EF <26% receiving 30 mg of pSDF-1 showed increased EF at 12 months (*p* = 0.01)	Patients receiving in 30 mg pSDF-1 showed a tendency for increased SV at 12 months (*p* = 0.09)	Unknown
AC6^5^	No difference vs. placebo	D4 +D5 (24) vs. placebo (14) Within-group: EF increase: *p* < 0.004 at 4 weeks; *p* = 0.16 at 12 weeks; no difference in placebo ETT increase: *p* = 0.06 at 12 weeks; placebo *p* = 0.03 at 12 weeks Between-group: LV –dP/dt decrease: *p* < 0.03 vs. placebo at 4 weeks No between-group difference in EF, ETT, or +dP/dt	D4 +D5 (24) vs. placebo (14) Within-group: reduced symptoms: *p* < 0.0005 at 12 weeks; no difference in placebo Between-group: no differences	Increased EF at 12 weeks in patients with non-ischemic HF (*p* = 0.025; post hoc) AC6 gene transfer tended to reduce HF hospitalization rate (*p* < 0.10)	FDA approved P3 trial for 2017

^a^ Treatment-emergent events occurring in ≥2% in both groups revealed a single difference: placebo-patients had an increased rate of ICD implantation.

^b^ See text^2^ for detailed explanation.

CV, cardiovascular.

#### *Efficacy* in CUPID

The 6 month primary endpoint was achieved in all three of the pre-specified analyses for the AAV1.*SERCA2a* high-dose group, including the group-level analysis in functional (6MWT, *p* = 0.14) and LV function/remodeling (End-systolic volume [ESV], *p* = 0.057) domains, the individual-level analysis (*p* = 0.052), and the outcome endpoint (using duration of cardiovascular hospitalizations, *p* = 0.08). The clinical event rate in all three AAV1.*SERCA2a* groups was delayed or reduced compared with placebo subjects. The hazard ratios (HR) at 12 months for recurrent clinical events (adjusted for correlated terminal events) for the low-, mid-, and high-dose AAV1.*SERCA2a* groups versus placebo were 0.40 (*p* = 0.11), 0.44 (*p* = 0.12), and 0.12 (*p* = 0.003), respectively (Table 2).

### CUPID 2

The CUPID-2 study^3^ was a Phase 2b randomized, double blind, multicenter study that enrolled patients with stable symptomatic HFrEF (EF ≤35%) (Table 1). Randomized patients had 50% non-ischemic etiology, and 77% had an ICD. Patients were randomized 1:1 to receive IC infusion of placebo (*n* = 122) or AAV1.*SERCA2a* (*n* = 121) at a dose of 10^13^ drp. The study product was delivered in proportion to myocardium perfused, targeting two-thirds of the dose to the anterolateral wall, and intravenous nitroglycerin was infused during test article delivery. The primary efficacy endpoint was time to recurrent events, defined as hospital admission because of HF or ambulatory treatment for worsening HF. After observing a lower event rate than anticipated in the first 101 patients, additional inclusion criteria of elevated N-terminal pro-B-type natriuretic peptide (NT-proBNP) or a recent HF-related hospital admission were added to the protocol.

#### Safety in CUPID-2

No group differences in serious adverse events were seen. There were 262 clinical events in the placebo group and 190 events in the AAV1.*SERCA2a* group, most of which were admission to hospital. Death occurred in 16% of subjects in the placebo group (18/20 deaths were of cardiovascular origin) and in 21% of the subjects in the AAV1.*SERCA2a* group (22/25 deaths were of cardiovascular origin) (Table 2).

#### Efficacy in CUPID-2

Patients were followed for at least 12 months after randomization (median 18 months). AAV1.*SERCA2a* did not improve time to recurrent events compared to placebo (104 vs. 128 events; HR = 0.93, *p* = 0.81). There were 104 recurrent and 36 terminal events reported in the AAV1.*SERCA2a* group compared to 128 recurrent and 29 terminal events in the placebo group. Most of the recurrent events were admission to hospital because of HF. There were no group differences in the primary endpoint or any of the secondary or exploratory efficacy endpoints (change in NYHA class, 6 min walk distance, symptoms, or NT-proBNP level). Nonstandard statistical methods were used for the CUPID study, and this may have contributed to the disparity in results between CUPID and CUPID 2 (Table 2).

### STOP-HF

#### Preliminary preclinical and nonrandomized clinical studies

SDF-1 is a factor that activates endogenous stem cells, which facilitates tissue repair particularly through vasculogenesis.^9,10^ The authors rationale was to use gene transfer of a DNA plasmid encoding human *SDF-1 (pSDF-1)* to increase tissue repair pathways including myocardial vasculogenesis and thereby improve function of the failing ischemic heart. Indeed, their group had shown in a pig HF model that endocardial injection of *pSDF-1* increased cardiac function and vasculogenesis 90 days after delivery.^10^

This strategy differs from the others reviewed in three ways. First, SDF-1, unlike SERCA2a and AC6, is a cytokine that has no direct effects on cardiac function, but operates via stimulating endogenous repair processes including angiogenesis. Second, a plasmid vector rather than a virus vector is used. Third, delivery is by direct injection into the heart rather than by IC delivery.

In an initial nonrandomized, open-label clinical trial,^11^ 17 subjects with symptomatic ischemia-based HFrEF (EF <40%) and an ICD received one of three doses (5, 15, or 30 mg) of *pSDF-1* delivered via endocardial catheter-based injection (15 injections of equally divided amounts of *pSDF-1* per patient). There was no placebo control in this initial trial, but improvements in 6 min walk distance and symptoms were seen in the groups that received 15 mg (*n* = 6) and 30 mg (*n* = 7) of *pSDF-1*. There were some procedure-related adverse events that were deemed to be at an acceptably low rate.

The STOP-HF trial^4^ was a Phase 2 blinded, placebo-controlled, multicenter study in which 93 subjects with ischemic etiology stable symptomatic HFrEF (EF <40%) and an ICD were randomized 1:1:1 to receive endocardial catheter-based injection of placebo or 15 or 30 mg of *pSDF-1* (Table 1). The primary endpoint was a composite of symptoms (Minnesota Living with Heart Failure Questionnaire) and 6 min walk distance evaluated before and 4 months after randomization. Echocardiography was used to assess LV dimensions and function, which were secondary endpoints. Enrollees had a mean EF of 28 ± 7%, and were 11 ± 9 months out from myocardial infarction.

#### Safety in STOP-HF

There were no product-related unanticipated serious adverse events among the 62 patients randomized to receive *pSDF-1* (Table 2).

#### Efficacy in STOP-HF

The primary endpoint of the trial was not met (*p* = 0.89), as the composite score of the 6 min walk distance and symptoms was increased in both placebo and treated cohorts 4 months after randomization. However, a pre-specified analysis indicated that patients with the lowest tertile of EF who received the higher dose of *pSDF-1* (30 mg) showed a 7% unit increase in EF compared with a 4% unit decrease in EF among placebo patients at 12 months (*p* = 0.01). The authors concluded that although the primary endpoint was not met, endocardial injection of *pSDF-1* was safe and possibly effective in patients with the lowest EF, which may be the target population in subsequent trials (Table 2).

### AC6 gene transfer trial

AC6 is a large (130 kD) protein that catalyzes the conversion of adenosine triphosphate (ATP) to cyclic adenosine monophosphate (cAMP). Myocardial cAMP levels are an important element in heart function. In studies conducted in pigs with HF, IC *AC6* gene transfer increased LV AC6 content, improved heart function, and reversed adverse LV remodeling.^12^

The benefits of AC6 appear to be independent of increased levels of cAMP through favorable actions on Ca2+ handling.^13,14^ These novel properties of AC6 differ from drugs that increase intracellular cAMP, such as milrinone, which reduce survival in HF. Indeed, increased cardiac AC6 content prolongs life in murine genetic cardiomyopathy.^15^ Finally, unlike milrinone, in preclinical studies, AC6 does not cause unbridled cAMP generation and reduces arrhythmias.^16^

The trial examined the safety and efficacy of IC delivery of Ad5.*hAC6* in patients with symptomatic HFrEF (EF ≤40%). This multicenter, double-blind, placebo-controlled, Phase 2 study^5^ randomized 56 subjects at a 3:1 ratio to receive IC Ad5.*hAC6* at one of five doses, ranging from 3.2 × 10^9^ to 10^12^ virus particles (vp) or placebo, along with concomitant infusion of IC nitroprusside (Table 1). The primary endpoints were change from baseline in treadmill exercise time in rest or dobutamine-stimulated EF by echocardiography and in LV pressure development (+dP/dt) or decline (–dP/dt) before and during dobutamine infusion during cardiac catheterization. Follow-up measurement of dP/dt was at 4 weeks; exercise testing and EF were reassessed 4 and 12 weeks after randomization. Pre-specified analyses included a key efficacy comparison on an intention-to-treat basis of the 14 placebo participants, with the 24 Ad5.*hAC6* participants receiving the highest two doses (D4 + D5).

Data were reported for 56 subjects who were randomized and monitored for up to 1 year. The 42 (75%) subjects who received Ad5.*hAC6* were well matched with the 14 (25%) who received placebo. Approximately half of each group had an ischemic etiology of HF.

#### Safety in AC6 gene transfer trial

No significant adverse events occurred during study product administration. Transient troponin I elevations (with normal CKMB and no chest pain or ECG changes) occurred the following day, leading to 1 day hospitalization in three participants, one of whom received placebo. Troponin I was increased compared with baseline levels on days 2 and 4 in both the placebo and D4 + D5 participants, with no group differences and no increases in creatine kinase MB. There was no evidence for hepatotoxicity in patients receiving Ad5.hAC6.

At 12 months, the percentage of participants with one or more serious adverse events was similar between groups. The HF admission rate was 9.5% (four episodes among 42 subjects) among the AC6 participants and 28.6% (four episodes among 14 subjects) among placebo participants (*p* < 0.10). There were two deaths, one in each group: (AC6, 2%; placebo, 7%; *p* = 0.40). There were two transplantations, both in AC6 participants (*p* = 0.9 vs. placebo). The overall rates of ICD events and episodes of non-sustained ventricular tachycardia showed no group differences (Table 2).

#### Efficacy in AC6 gene transfer trial

Twenty-one subjects who received the highest two doses of Ad5.*hAC6* (D4 + D5) had a significant increase in EF at 4 weeks (29.7–36.3%, mean increase of 6.0 EF units; *p* < 0.004) but not at 12 weeks (29.7–34.2%, mean increase of 3.0 EF units; *p* = 0.16). The increase in EF at 4 weeks in AC6 participants was dose related (*p* < 0.04). The 14 subjects who received placebo had an increase from baseline EF that was not statistically significant at 4 weeks (29.6–33.7%, mean increase of 4.1 EF units; *p* = 0.08) or at 12 weeks (29.6–31.6%, mean increase of 0.8 EF units; *p* = 0.31).

Treadmill performance did not increase in the 17 D4 + D5 participants who were able to exercise at 4 weeks or in the 13 placebo participants (from 416 to 446 s; *p* = 0.46). Treadmill time increased to 501 s at 12 weeks in both groups, which was a significant change from baseline in the 12 placebo subjects (*p* = 0.03) but not in the 17 D4 + D5 subjects (*p* = 0.06).

Basal (unstimulated) LV peak –dP/dt at 4 weeks improved in 21 D4 + D5 subjects (−39 mmHg/s) but worsened after randomization in placebo subjects (+93 mmHg/s change), and this between-group difference was significant (*p* < 0.03). There was no group difference in dobutamine-stimulated LV peak +dP/dt or LV peak –dP/dt.

Neither group had a change in HF symptom score at 4 weeks. At 12 weeks, there was a reduction in symptom score in D4 + D5 participants (from 28.8 to 20.5; *p* = 0.0005) but not in placebo participants (from 30.8 to 23; *p* = 0.16). Anti-Ad5 titers ranged from <1:18 (undetectable) to >1:4,608. There was no association between pre-randomization anti-Ad5 titer and any efficacy endpoint (Table 2).

The investigators concluded that this one-time administration of Ad5.*hAC6* safely increased LV function beyond standard HF therapy and that larger trials were warranted.

### Safety of clinical gene transfer in HFrEF

In each of these trials, gene transfer was performed with an acceptable safety profile (Table 2). The delivery technique was not associated with an increased rate of adverse events. Subjects had an uneventful subsequent early course, with only low levels of troponin-I elevation without clinical sequalae noted in both the placebo and Ad5.*hAC6* subjects in the AC6 trial. There was no evidence of adverse consequences of gene transfer at 1 year follow-up in CUPID-2, STOP-HF, and Ad5.*hAC6*.

The genes delivered in these trials were anticipated to improve the function of the failing heart in different ways. To promote the extent of IC vector delivery and thereby reduce vector dose, simultaneous intravenous nitroglycerine^17^ or IC nitroprusside^18^ were used. Arrhythmias were a theoretical concern, and in these trials most of the subjects had an ICD in place. However, there was no increase in arrhythmias seen in the subjects who received gene transfer in these trials, in particular there was no increase in arrhythmic clinical events compared with subjects who received placebo (Table 2).

The safety profile of clinical gene transfer in this high-risk patient population is a major finding. Recognizing the early and mixed results in terms of efficacy in these studies, safety supports further investigations using gene transfer.

## Conclusions

The favorable safety profile seen now in four randomized clinical trials of gene transfer in patients with HFrEF has paved the way for further progress in this field. Although the experience with these agents is limited, clinically evident adverse immunologic responses, arrhythmias, and other adverse events have not been as high as anticipated. The clinical course of subjects receiving gene transfer has been as good as or better than that of subjects receiving placebo. Remaining challenges include refinement of endpoints, optimal dosing, selection of transgene, and the persistence of benefits.

The promising potential efficacy of AAV1.*SERCA2a* seen in the initial CUPID was not borne out by the larger CUPID 2 study. The STOP-HF study showed potential benefits in patients with the lowest EF, and additional trials may be planned. The current work with Ad5.*hAC6* has shown potential benefit at higher doses. A larger study will be required to confirm the preliminary indications of improvement in EF and the reduction of clinical adverse events.
